# Gut microbiota-host interactions and juvenile idiopathic arthritis

**DOI:** 10.1186/s12969-016-0104-6

**Published:** 2016-07-22

**Authors:** Miika Arvonen, Lillemor Berntson, Tytti Pokka, Tuomo J Karttunen, Paula Vähäsalo, Matthew L Stoll

**Affiliations:** Department of Pediatrics, Kuopio University Hospital, Kuopio, Finland; Medical Research Center, Oulu University Hospital and University of Oulu, Oulu, Finland; PEDEGO Research Unit, University of Oulu, Oulu, Finland; Department of Women’s and Children’s Health, Uppsala University, Uppsala, Sweden; Department of Children and Adolescents, Oulu University Hospital, Oulu, Finland; Cancer and Translational Medicine Research Unit, University of Oulu, Oulu, Finland; Department of Pathology, Oulu University Hospital, Oulu, Finland; Department of Pediatrics, University of Alabama at Birmingham, CPP N 210 M, 1600 7th Avenue South, Birmingham, AL 35233 USA

**Keywords:** Juvenile arthritis, Microbiota, Antibiotics

## Abstract

**Background:**

Juvenile idiopathic arthritis is the most common form of chronic arthritis in children. There is mounting evidence that the microbiota may influence the disease.

**Main body:**

Recent observations in several systemic inflammatory diseases including JIA have indicated that abnormalities in the contents of the microbiota may be factors in disease pathogenesis, while other studies in turn have shown that environmental factors impacting the composition of the microbiota, such as delivery mode and early exposure to antibiotics, affect the risk of chronic inflammatory diseases including JIA. Microbial alterations may predispose to JIA through a variety of mechanisms, including impaired immunologic development, alterations in the balances of pro- versus anti-inflammatory bacteria, and low-grade mucosal inflammation. Additional confirmatory studies of microbiota aberrations and their risk factors are needed, as well as additional mechanistic studies linking these alterations to the disease itself.

**Conclusions:**

The microbiota may influence the risk of JIA and other systemic inflammatory conditions through a variety of mechanisms. Additional research is required to improve our understanding of the links between the microbiota and arthritis, and the treatment implications thereof.

## Background

The last decade has witnessed an explosion of research into the causes and consequences of alterations in the microbiota. One condition that has recently become the subject of interest in this respect is juvenile idiopathic arthritis (JIA). JIA is a heterogeneous autoimmune disease comprising seven categories, several of which have distinctive clinical and genetic features [1]. Some of the categories are related to adult counterparts, for which there is also accumulating evidence of a role of the microbiota. In this review, we discuss the nature of the microbiota in JIA, factors that may predispose to dysbiosis, and mechanisms by which an altered microbiota might predispose to arthritis.

## Querying the microbiota

The oldest method of identifying bacteria is culture. While this remains an important tool in clinical medicine, it is an ineffective means of identifying the contents and relative abundances of complex communities of organisms, many of which are difficult if not impossible to culture [2]. Until recently, a widely used tool consisted of amplification of the 16S ribosomal DNA gene followed by gel electrophoresis. This allowed for visual assessment of differences in the contents of the microbiota, but did not itself provide information on the identity let alone the function of any of the organisms. Today, technology permits sequencing of whole communities. A detailed discussion of sequencing technologies and associated informatics tools are beyond the scope of the review and are available to the interested reader [3]. Two major sequencing technologies are in use today: amplicon-based, which typically consists of PCR amplification followed by sequencing of the 16S ribosomal DNA region; and whole genome sequencing (WGS), in which every bit of microbial DNA is sequenced. Sequencing of the 16S ribosomal DNA region takes advantage of the immense variability among bacteria contained in this one region. Compared to WGS, this approach has the advantage of lower cost and relatively easier analytic tools; in contrast, WGS can more readily identify bacteria at the species and even strain level and also provides direct functional information on the bacteria.

## Intestinal microbiota in JIA

Two studies have evaluated the contents of the fecal microbiota in children with JIA. In a recent Finnish study [4], microbiome profiles of fecal samples of 30 untreated children with JIA (mostly with oligoarticular and rheumatoid factor-negative polyarticular JIA) were analysed with 16S region-based sequencing profiling, and were compared to fecal samples of 27 healthy controls. The proportion of bacteria belonging to the phylum Firmicutes was significantly lower in children with JIA compared to controls, with a compensatory increase in the Bacteroidetes phylum. At the genus level, increased *Bacteroides* was observed among the children with JIA. Similar abnormalities have also been reported in children with or at risk for type 1 diabetes mellitus [5–7]

A similar, albeit not statistically significant (21 % versus 11 %, *P* = 0.150) increase in the *Bacteroides* genus was also observed in the feces of a cohort of 25 children with enthesitis-related arthritis (ERA) as compared to 13 healthy control subjects [8]. This study also showed elevated levels of *Akkermansia muciniphila* in a subset of patients, but none of the controls [8]. In contrast, levels of *Faecalibacterium prausnitzii* were reduced in the ERA patients. This latter finding is consistent with observations in both pediatric and adult inflammatory bowel disease (IBD) [9].

## Contribution of perinatal factors in the gut microbiome

Genetic and environmental factors influence the development of the microbiota. A discussion of the genetic factors is beyond the scope of this review, which is geared towards potentially modifiable influences. Among those, early life factors such as mode of delivery, lactation and early exposure to antibiotics influence the type of bacteria colonizing intestinal mucosa and maturation of mucosal immunity [10–12]. Vaginal delivery promotes the infant gut to colonize with *Bifidobacteria*, which is associated with stimulation of tolerogenic immune responses [13, 14]. In contrast, infants delivered by C-section harbour bacterial communities found on the mother’s skin surface such as Staphylococcus, Corynebacterium, and Propionibacterium spp, and higher counts of IgA, IgG, IgM secreting cells during first year of life [15–17]. Interestingly, C-section delivery is associated with an increased risk of development of multiple chronic inflammatory conditions, including food allergy, inflammatory bowel disease, type 1 diabetes and JIA [18–20].

Another perinatal variable that influences the microbiota is mode of feeding. As with mode of delivery, several observational studies have demonstrated alterations in the fecal microbiota of children bottle-fed compared to those who are nursed. Just as vaginally born infants have higher *Bifidobacterium* as compared to C-sectioned infants, nursed infants appear to have higher abundance of the same species, as well as increased abundance of *Lactobacilli* and *Streptococci*, two normal components of the infant fecal microbiota [21]. There is some data indicating that bottle feeding is associated with an increased risk of autoimmune disease like ankylosing spondylitis [22] and type 1 diabetes [23]. Similarly, breast-feeding appears to be protective against JIA, as evidenced by either increased likelihood or duration of breast-feeding among JIA children compared to controls [24–26].

## Antibiotic use, microbiome alteration and risk of JIA

Another environmental influence on the microbiota is antibiotic usage. That antibiotics have a short-term effect on the contents of the microbiota is self-evident. Numerous studies have evaluated whether this effect is sustained over time (Table 1.). The methods of assessment of the faecal samples were variable including culture, gel electrophoresis, and amplification followed by sequencing of 16S ribosomal DNA, tools which as discussed above have increasing sensitivity in respective order to identify the complexity of bacterial organisms present in a sample. It appears that in many instances antibiotics do indeed affect the microbiota long-term, even up to two years in one study [27]. To some extent, it appears that antibiotics that target anaerobic organisms were more likely than others to have a lasting impact. However, ciprofloxacin did as well, particularly when patients were exposed to multiple courses [28]. In addition, a cross-sectional study in children indicated that prior exposure to macrolide antibiotics had substantial and long-lasting effects on the microbiota [29].

**Table 1:** Summary of human studies evaluating long-term changes to the microbiota following exposure to antibiotics

Study	Antibiotic	Patient population	Comparison Group	Habitat	Method of assessment	Duration of follow-up	Results
De la Cochetiere (2005) [72]	Amoxicillin x 5 days	6 adults	None	Feces	TTGE of 16S rDNA amplicons	Two months	After two months, profiles were >90 % similar to baseline in 5/6 subjects.
Dethlefsen 2011 [28]	Two courses of ciprofloxacin x 5 days	3 adults	None	Feces	Sequencing of 16S rDNA	10 months	Altered community composition in 3/3, although there was more variability between subjects vs before and after abx.
Dethlefsen 2008 [73]	One course of ciprofloxacin x 5 days	3 adults	None	Feces	Sequencing of 16S rDNA	30 days	Samples returned to baseline at the community level after 30 days, although individual taxa failed to recover.
Fouhy 2012^a^ [74]	One course of ampicillin and gentamycin	9 full-term neonates under age 2 days	9 full-term neonates	Feces	Sequencing of 16S rDNA	8 weeks	Decreased evenness and richness; alterations in multiple genera. Of note, 9/9 controls but only 4/9 patients were delivered vaginally
Jakobsson 2010 [75]	One course of metronidazole and clarithromycin x 7 days	3 adults	3 adults	Throat and feces	Sequencing of 16S rDNA and T-RFLP	4 years	General recovery of loss of diversity in both habitats. However, long-lasting effects at the taxonomic level were seen, particularly in the throat.
Jernberg 2007 [27]	One course of clindamycin x 7 days	4 adults	4 adults	Feces	T-RFLP and rep-PCR on *Bacteroides*	2 years	Decreased number of bacteroides clonal types in exposed subjects
Lode 2001^b^ [76]	Linezolid x 7 days	12 adults	None	Feces	Culture and identification	35 days	No lasting effect
Lode 2001^b^ [76]	Amoxicillin / clav x 7 days	12 adults	None	Feces	Culture and identification	35 days	No lasting effect
Mangin 2012 [77]	Amoxicillin / clav x 5 days	18 adult men	None	Feces	qPCR for *Bifidobacterium* and PCR-TTGE	64 days	No difference in total bifidobacteria; however, similarity to baseline dropped to 50 % rapidly and never reached 60 %.
Savino 2011 [78]	Ceftriaxone x 5 days	26 full-term breast-fed infants	None	Feces	Culture	20 days	No changes noted in counts of enterobacteriaceae, enterococci, lactobacilli, or total bacteria
Vervoort 2015^c^ [79]	Nitrofurantoin x 3 – 15 days	Five or eight subjects	Four or five subjects	Feces	Sequencing of 16S rDNA	28 days	Only transient differences in the frequency of the phyla.

^a^The duration of treatment was not specified. ^b^This Lode study was a crossover design with a 35 days washout, in which half received amoxicillin / clavulonic acid first and the other half received linezolid first. ^c^The methods said five pts and four controls, but the table said 8 and 5, respectively. No information on the age or sex of the subjects. Abbreviations: *qPCR* quantitative PCR, *rDNA* ribosomal DNA, *rep-PCR* repetitive sequence-based PCR, *RFLP* restriction fragment length polymorphisms, *T-RFLP* terminal restriction fragment length polymorphism, *TTGE* temporal temperature gradient gel electrophoresis

Two registry-based case controls studies have evaluated whether antibiotic use affects subsequent risk of JIA. Horton et al. [30] identified medical records from a database of 550 general practices in the United Kingdom, identifying 152 children with JIA and 1520 matched controls. Arvonen et al. (2015) collected data from three Finnish national registers to identify 1298 children with JIA and 5179 matched controls [31]. Both studies found a significant association between antibiotic use and subsequent JIA (Table 2), both also reporting a dose-dependent relationship; the Finnish registry as well showed that early exposure (<24 mos) was associated with risk of developing JIA (OR 1.4, 95 % CI 1.2–1.6). The UK study also found that this relationship held even after adjusting for infections; this was not addressed in the Finnish register-based study. The two studies did differ with respect to one critical finding: whether the antibiotics differ in their association with JIA. The UK study grouped antibiotics into those with versus without anaerobic coverage (Table 2), finding that exposure to both categories had a similar effect on the risk of subsequent JIA. The rationale for such a grouping is that the vast majority of enteric organisms are either facultative or obligate anaerobes, and as noted above, antibiotics with primarily anaerobic coverage appear to have a longer-lasting effect on the gut microbiota as opposed to antibiotics that primarily target aerobic organisms. Although not initially published in the Finnish study, we performed for the purposes of this a review conditional regression analysis of the previously published data using the same definition of anaerobic antibiotics used in the UK study. After adjustment for total number of courses of antibiotics, children exposed only to aerobic antibiotics had a non-significantly increased risk (OR = 1.2, *p* = 0.122), while those exposed to only anti-anaerobic antibiotics had a significantly increased risk (OR = 1.3, *p* = 0.021), and those exposed to both categories had the highest risk (OR = 1.4, *p* = 0.003). That is, children exposed to a variety of different antibiotic categories appear to be at higher risk than those exposed to a more limited repertoire of antibiotics, even after adjusting for total number of courses, suggesting that the greater the overall perturbation of the microbiota, the greater the risk of JIA.

**Table 2:** Characteristics of the material and results in studies on exposure to antibiotics and risk of juvenile idiopathic arthritis by Horton et al. [30] and Arvonen et al. [31]

	Horton et al.		Arvonen et al.	
	United Kingdom		Finland	
	Cases = 152, Controls = 1520		Cases = 1298, Controls = 5179	
Risk of later development of JIA after exposure to	OR (95 % CI)^a^	P	OR (95 % CI)^b^	P
Any antibiotics	2.1 (1.2 to 3.5)	0 .007	1.6 (1.3 to 1.9)	<0.001
Anaerobic antibiotics only^c^	1.6 (1.0 to 2.6)	0.040	1.3 (1.04 to 1.7)	0.021
Non-anti-anaerobic only^c^	1.6 (1.1 to 2.3)	0.009	1.2 (0.9 to 1.7)	0.216
Both non-anti-anaerobic and anti-anaerobic antibiotics^c^	NA	NA	1.4 (1.1 to 1.8)	<0.001
Dose response	yes		yes	

^a^Models adjusted for matching, any infection, and any personal autoimmune disease (AID)

^b^Model adjusted for the number of antiobiotic regiments before index day

^c^For this analysis, anti-anaerobic antibiotics were broad spectrum penicillins, clindamycin, metronidazole, and tetracyclines (including doxycycline); aerobic antibiotics were cephalosporins, levaquines, macrolides, and sulfonamides

## Mechanisms by which the microbiota might predispose to JIA

### Dysbiosis: excessive “bad” or insufficient “good” bacteria

The most straightforward explanation by which the microbiota might predispose to JIA pertains to its contents. Although true pathogens are infrequently identified in most 16S studies performed in developed nations, certain bacteria appear to have the capacity to promote an inflammatory process, while others appear to be protective. For example, Scher and colleagues identified abundant *Prevotella copri* in many newly diagnosed rheumatoid arthritis (RA) patients, demonstrating as well that this bacteria could directly trigger inflammatory responses in mice [32]. Conversely, as noted above, Stoll and colleagues reported decreased abundance of *F. prausnitzii* in children with ERA [8]. This particular species is generally considered to have anti-inflammatory effects through production of short-chain fatty acids (SCFAs) such as butyrate [33] or by direct effects on cytokine production [34]. SCFAs serve as major sources of energy for the intestinal enterocytes and also regulate the differentiation of T cells, promoting a regulatory phenotype [35, 36]. Additionally, as noted above, both 16S studies of children with JIA demonstrated increased *Bacteroides* genus in children with JIA compared to controls. The potential for this genus to demonstrate pathogenicity in arthritis was illustrated by animal models of arthritis, in which the disease is abrogated in the germ-free state but present upon re-introduction of *Bacteroides* genus [37, 38]. Thus, certain bacteria can have direct inflammatory or anti-inflammatory effects promoting or inhibiting the development of inflammatory disease.

There is, however, limited direct evidence in JIA that the microbiota is directly responsible, or that changes in the microbiota can affect changes in the disease. Zhang et al. (2015) identified differences in the oral and gut microbiota in treatment-naïve RA patients, finding as well partial normalization following induction of disease-modifying therapy as well as pre-treatment differences in good versus poor responders to therapy [39]. This has not been studied comprehensively in children with JIA. Berntson et al. reported on a child with polyarticular JIA refractory to multiple medicines, in whom a beneficial clinical response to exclusive enteral nutrition was accompanied by elevation of Firmicutes/Bacteroidetes ratio during the treatment, although the microbiota changes were not conclusively demonstrated to be responsible for the clinical improvement [40]. Interestingly, in some other autoimmune diseases, correlation of microbiome composition and clinical course have been documented. A study of children at risk for type 1 diabetes showed that changes in the composition and diversity of the microbiota preceded development of clinical disease [7]. Likewise, a study of pediatric IBD patients revealed that disease activity was associated with reduced microbial richness, abundance of butyrate producers, and relative abundance of Gram-positive bacteria [41].

Importantly, bacteria need not be the only type of microorganism that can affect immune responses and autoimmune diseases; helminths may also modify intestinal microbiome homeostasis [42]. In addition, helminth colonization and molecules from helminths have been demonstrated to trigger regulatory pathways and attenuate the course of experimental arthritis [43]. Helminths have even been studied as a novel treatment approach for IBD [44]

### Immune programming

A second mechanism by which the microbiota could impact the risk of inflammatory disease may pertain to the ontogeny of the microbiota, rather than its nature in the mature state. A dramatic illustration of this possibility comes from studies of germ-free animals, in which the small intestinal mucosal immune system fails to develop properly. For example germ free mice develop fewer Peyer’s patches, germinal centers and lymphoid follicles in small intestinal mucosa and express reduced numbers of Th17 cells in small intestinal lamina propria [45]. Moreover, there may be a critical window of time during which the proper microbiota must be present for optimal immunologic development, as recently proposed by Blumberg and colleagues [46]. This possibility was reported by Cahenzli et al. (2013), who confirmed previous findings of elevated IgE levels in germ-free mice [47], a murine counterpart to the hypothesis that today’s cleaner environments may be associated with the increased incidence of autoimmune conditions such as atopy and IBD [48]. A key finding in the study by Cahenzli was that mice exposed to normal microbiota prior to 35 days of life had normal IgE levels, while those whose exposure took place beyond that point had high levels. Similarly, colonization of young, but not old, germ-free mice with a normal microbiota abrogated the accumulation of invariant natural killer T cells in the colonic lamina propria and lung [49]. This concept of a window of opportunity may also explain why *Bacteroides* appears to be associated with autoimmunity in young [4, 7, 50], but not adult [32, 51], subjects; Vatanen et al. [52] proposed that the lipopolysaccharide (LPS) tail of *Bacteroides* is less immunostimulatory as compared to the LPS tail of bacteria such as E. coli, and that early exposure to *Bacteroides* in countries such as Finland predisposes to autoimmunity due to excessive innate immune tolerance towards LPS in general. Thus, it is plausible that antibiotics in young children, in whom the microbiota is undergoing rapid changes to a more mature state, would have longer lasting effects on both the contents of the microbiota as well as subsequent immunologic function, as compared to antibiotic exposure in older children or adults. If so, this underscores the necessity of avoiding unnecessary usage of antibiotics.

### Aberrant microbe-specific systemic immune responses in JIA

A given bacterial species need not be present in abnormal quantities to cause problems; it may also lead to inflammation by being a target of the immune system. Antibodies against flagellated bacteria are associated with and poor prognostic factors for Crohn’s Disease [53]. There is also contradictory evidence as to whether these antibodies are associated with spondyloarthritis [50].

Pediatric patients may have different immunologic targets. Stoll et al. demonstrated that in children with ERA [8], serum IgA against *B. fragilis* was directly proportional to fecal *Bacteroides* abundance, while controls had the opposite relationship, which they took to indicate that patients had a more pathogenic response against this organism. Additionally, Singh et al. (2011) demonstrated increased T cell responses to the *Salmonella* outer membrane protein in ERA patients compared to controls [54].

These findings of aberrant immunity to intestinal or commensal organisms are not necessarily limited to children with ERA. Children with Cyclic Citrullinated Peptide (CCP) antibody positive, as compared to negative, JIA demonstrated elevated levels of antibodies against *Porphyromonas gingivalis, Prevotella intermedia,* and *Fusobacterium*. This study also showed an association between high anti-bacterial antibodies levels and clinical symptoms of gingival irritation [55]. These data are generally consistent with studies in the adult counterpart, RA [56]. The presence of antibodies directed against specific microbial agents does not necessarily indicate that the antibodies themselves are pathogenic. Antibodies reflect activity of T_h_ cells, which may themselves be the pathogenic cells. This appears to be the case in IBD, where adoptive transfer of flagellin-specific CD4+ T cells into immunodeficient mice results in colitis [57]. Similarly, disease in the HLA-B27 transgenic rat model requires the presence of a thymus [58]; while the nature of any antigens in this model are not known, the absence of disease in the germ-free state suggests the possibility of microbial antigens [59]. The causes of such aberrant immunity as well as the mechanisms by which microbe-specific immunity may contribute to JIA are ripe for further exploration.

### Local immune responses and mucosal integrity in JIA

**Fig. 1:**
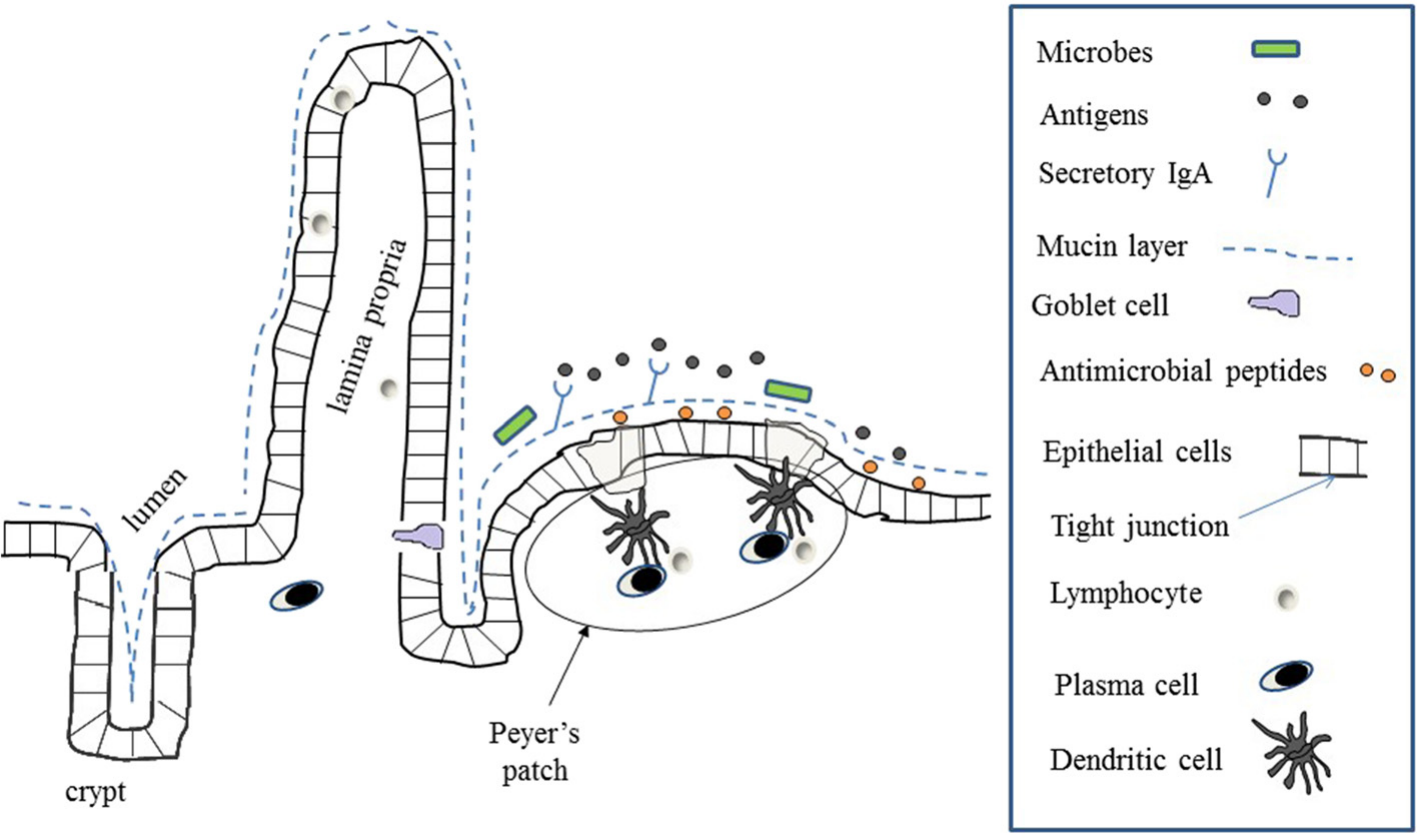
The structure of intestinal mucosal defense and antigen sampling. Primary defense against penetration by luminal microbes is primarily provided by secretory IgA, mucin and antimicrobial peptides. In addition, single layered intestinal epithelial cell are anchored to each other by tight junctions. Goblet cells scattered among the epithelial lining produce mucin, which represents a physical barrier against bacterial access to epithelial cells. Secretory IgA attaches to luminal antigens and protects against invasion of pathogens inhibiting the penetration of harmful antigens. On the epithelial side of the mucin layer, antimicrobial peptides neutralize bacteria that have penetrated through the mucin layer. The Peyer’s patch also contains a specific type of enterocytes, M-cells, which periodically sample the luminal contents, transcytosing luminal antigens. Antigens that have broken through the epithelial barrier to the basolateral lamina propria generate inflammatory responses, while those presented to Peyer’s patches by periodic sampling typically generate regulatory responses [80, 81]. Additionally, T cells activated in mesenteric lymph nodes (not shown) express intestinal homing receptors such as the integrin α4β7, which guide the T cells back to the intestinal mucosa, where they can participate in protective or inflammatory immune responses.

The intestinal microbiota may also have local effects on mucosal integrity and intestinal immunity. The intestinal mucosa limits access of gut bacteria to the lymphoid tissues, thereby preventing dysregulated activation of the local innate and adaptive immune system [60]. As discussed above, increased *Bacteroides* and *Akkermansia muciniphila* have been reported in children with JIA; increased abundance of *Bacteroides* has also been observed in children with type 1 diabetes [5, 6], as well as in animal models of spodyloarthritis [61, 62]. Multiple species in the *Bacteroides* genus, as well as *Akkermansia Muciniphila*, degrade mucin [63, 64], an important component of primary mucosal defense (Fig. 1). It is plausible that mucin degradation can increase access of the bacteria to the intestinal immune system, promoting an inflammatory process, as proposed by Tailford and colleagues [65]. Along those lines, increased intestinal permeability has been identified both in children with JIA [45] and in adults with ankylosing spondylitis [66].

Intestinal inflammation in children with JIA has been evaluated mostly in the ERA category. Studies of unselected children with ERA have demonstrated increased intestinal inflammation by colonoscopy [75], leukocyte scintigraphy [76], and fecal calprotectin [67]. Children with oligoarticular and polyarticular JIA may also have intestinal immune dysregulation, although studies are limited to children with gastrointestinal complaints. Specifically, Arvonen and colleagues reported “low grade” intestinal mucosal alterations such as increased numbers of small intestinal intraepithelial γδ + T cells and cytotoxic lymphocytes, and increased HLA-DR expression in ileal mucosa [68–70], the latter correlating with measures of JIA disease activity. Pichler et al. (2016) also identified increased eosinophilic gastrointestinal infiltrations in a cohort of JIA patients with gastrointestinal symptoms [71] increased eosinophilic gastrointestinal infiltrations. Thus, it is possible that abnormalities in mucosal immunity is a widespread phenomenon in children with JIA. However, some of these findings have not directly been linked to the microbiota.

## Conclusions

Children with multiple categories of JIA have an altered intestinal microbiota, with the characteristics of microbiota sharing some features linked with other autoimmune diseases such as type 1 diabetes [5, 6] and IBD [9]. In addition, the immunologic responses to the microbiota are altered in at least ERA and RF+ JIA, and aberrant intestinal immunity appears to be present in multiple JIA categories. Several of the identified risk factors of JIA, including antibiotic use, C-section delivery, and possibly infant feeding practice, may all exert their role via alterations in the intestinal microbiota, potentially at a critical window of mucosal immunologic development. In the future, the role of immune regulatory function of helminths should also be studied, since they can attenuate the course of experimental arthritis [43]. As we learn more about factors that influence the development of the microbiota as well as the mechanisms by which the microbiota might contribute to inflammation, we may develop novel tools to prevent or even treat JIA.

## Abbreviations

ERA, enthesitis-related arthritis; IBD, inflammatory bowel disease; JIA, juvenile idiopathic arthritis; RA, rheumatoid arthritis; RF, rheumatoid factor; SCFAs, short-chain fatty acids
